# Molecular detection and characterization of spotted fever group *Rickettsia* and *Anaplasma* in ticks from Pakistan

**DOI:** 10.1017/S0031182025100358

**Published:** 2025-05

**Authors:** Khan Sadia Salim, Ahmed Haroon, Benedict S. Khoo, Evan J. Kipp, Abid Ali, Davide Sassera, Muhammad Umair Aziz, Jonathan D. Oliver

**Affiliations:** 1 Department of Biosciences, COMSATS University Islamabad (CUI), Islamabad, Pakistan; 2 Division of Environmental Health Sciences, School of Public Health, University of Minnesota, Minneapolis, Minnesota, USA; 3 Department of Veterinary and Biomedical Sciences, University of Minnesota, St. Paul, Minnesota, USA; 4 Department of Zoology, Abdul Wali Khan University Mardan, Mardan, Pakistan; 5 Department of Biology and Biotechnology ‘L.Spallanzani’ University of Pavia, Pavia, Italy; 6 Department of Infectious Diseases and Public Health, Jockey Club College of Veterinary Medicine and Life Sciences, City University of Hong Kong, Kowloon, China

**Keywords:** *Anaplasma*, Pakistan, *Rickettsia*, ticks

## Abstract

The present study was designed to report the prevalence of spotted fever group *Rickettsia* and *Anaplasma* in ticks from Pakistan. To address this knowledge gap, ticks were collected from October 2019 to November 2020 from livestock hosts. Three hundred ninety ticks from Punjab, Khyber Pakhtunkhwa and Islamabad were investigated for the presence of *Rickettsia* and *Anaplasma*. The collected ticks were subjected to molecular studies for detection and characterization of spotted fever group *Rickettsia* and *Anaplasma* in ticks from Pakistan. PCR amplification of the *ompA* gene was used for detection of *Rickettsia* and portions of the *16S* rDNA gene for detection of *Anaplasma.* Nine species of ticks were tested. Of the 390 ticks tested, 7 (2.58%) ticks were positive for *Rickettsia. Rickettsia spp.* were detected in *Haemaphysalis punctata, Hyalomma anatolicum, Hyalomma scupense, Rhipicephalus microplus* and *Rhipicephalus sanguineus.* Unknown *Rickettsia* was detected in *Hy. scupense*. Fifty-seven (14.6%) ticks were also positive for *Anaplasma spp. Anaplasma ovis* was detected in *Hy. anatolicum, Hy. scupense, Hy. excavatum, Rhipicephalus decoloratus, R. microplus and R. sanguineus. Anaplasma marginale* was detected in *Hy. anatolicum, Hy. scupense, R. microplus, R. decoloratus* and *R. sanguineus*. The *Anaplasma* sequences obtained from this experiment were 99–100% similar to those of the documented strains. This study provides information and confirms the presence of spotted fever group *Rickettsia* and *Anaplasma spp.* in different tick species. It also highlights the need for control programs to prevent health risks. Further investigation to determine the prevalence and disease burden of these pathogens in Pakistan is necessary.

## Graphical abstract

**Figure:**
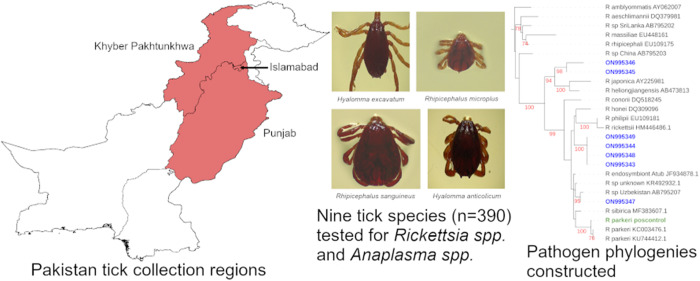

## Introduction

The livestock sector of Pakistan comprises primarily farmhouses that supply meat, leather and milk (Ashraf et al., 2020). Animals and their products are the main source of income in rural areas (Irshad et al., 2010; Rehman et al., 2017a), and livestock production represents 13.4% of GDP, the country’s largest agricultural sector (Rehman et al., 2017b). Livestock production is dominated by small farmers following traditional methods of production, with 94% of farms owning fewer than 10 cattle or buffaloes, with these farms representing 67% of Pakistan’s cattle holdings (Horst and Watkins, 2022). Infestation by ecto- and endoparasites substantially impacts livestock production in Pakistan (Sajid et al., 2008; Shahzad et al., 2013; Jabbar et al., 2015). For example, a study in Faisalabad found that tick infestation of cattle resulted in a morbidity rate of 43.7% and was the most prevalent disease condition reported (Ashfaq et al., 2014). Ticks also transmit many pathogens to animals and humans and contribute to the problem of livestock diseases (Parola and Raoult, 2001; Parola et al., 2013; Luce-Fedrow et al., 2015).

Tick-borne pathogens that affect humans are zoonotic in that they naturally circulate between ticks and vertebrate animal hosts with humans usually representing dead-end hosts. In Pakistan, most research into human tick-borne disease has focused on Crimean-Congo hemorrhagic fever virus (CCHFV), an endemic pathogen with a reported mortality rate as high as 40.7% in symptomatic cases (Umair et al., 2020). Although rickettsial pathogens that cause human disease are known to be present in Pakistan (Ullah et al., 2023), the distribution and diversity of these pathogens have been little studied representing a gap in the existing literature.

*Rickettsia* and *Anaplasma* (order Rickettsiales) are obligate intracellular bacteria. They are vector-borne pathogens mainly transmitted by ticks and cause diseases in humans and animals globally (Parola et al., 2013). There are four major groups of genus *Rickettsia* and, among them, typhus group and spotted fever group (SFG) are associated with human diseases. Studies have recorded more than 25 species of SFG *Rickettsia* (Gillespie et al., 2008; Thu et al., 2019) (SFGR) with worldwide distribution. At present, 8 species of *Anaplasma* including *A. marginale, A. phagocytophilum* and *A. ovis* are recognized (Dumler et al., 2001; Tate et al., 2013; Li et al., 2015; Silaghi et al., 2017).

Anaplasmosis has been reported in wild and domestic ruminants and is caused by members of genus *Anaplasma*, which infect blood cells in animals. *Anaplasma ovis* and *A. marginale* cause pathogenicity in small and large ruminants, respectively (Kocan et al., 2004; Liu et al., 2012)*. Anaplasma ovis* causes infections in sheep, deer and goats, whereas *A. marginale* causes anaplasmosis in cattle (Dumler et al., 2001; Ashraf et al., 2013, 2020; Seong et al., 2015). Occasionally, *A. ovis* has also been reported as the causative agent of human infections (Chochlakis et al., 2010; Hosseini-Vasoukolaei et al., 2014). Tick species from the genera of *Rhipicephalus, Dermacentor, Hyalomma, Ixodes* and *Argas* have been reported to transmit *Anaplasma* spp. (Kocan et al., 2004; Hairgrove et al., 2015; Jabbar et al., 2015; Battilani et al., 2017). Clinical findings of anaplasmosis in animals range from subclinical in < 1 year old to severe and often fatal in older cattle, and are characterized by anaemia, rapid loss of condition, reduced milk production, anorexia and abortion in pregnant animals (Aubry and Geale, 2011; Jaswal et al., 2015).

Pathogenic SFGR have been reported from ticks all over the world (Piotrowski and Rymaszewska, 2020; Zhang et al., 2023), and they are among the oldest known vector-borne diseases (Parola et al., 2005). SFGR have been insufficiently studied in Pakistan although previous studies have found that rickettsiae, including known pathogenic species, circulate in Pakistani ticks and livestock (Karim et al., 2017; Ali et al., 2022a, 2022b; Zeb et al., 2024). The aim of the present study was to improve understanding of the diversity, distribution and prevalence of SFGR and *Anaplasma* spp. in ticks from three provinces of Pakistan and to describe the phylogenetic relationships of these pathogens to existing records.

## Materials and methods

### Tick sampling and identification

Ticks from Punjab, Khyber Pakhtunkhwa and Islamabad were investigated. The majority of cattle, buffaloes and goats in the Pakistan are reared in Punjab and Khyber Pakhtunkhwa provinces (Pakistan Bureau of Statistics, 2006). In a previous study, ticks were collected from 1325 cattle (*Bos indicus; B. taurus*), 127 sheep (*Ovis aries*), 89 buffaloes (*Bubalus bubalis*) and 539 goats (*Capra hircus*; Khan et al., 2022). Of these, 1129 animals were infested with ticks. Ticks were preserved in 70% ethanol and male ticks were identified morphologically. In total, 390 individual male ticks representing 10 species were selected for DNA extraction and testing for the presence of *Rickettsia* and *Anaplasma*. Further details on tick species identification and animal infestation prevalence and risk factors can be found in (Khan et al., 2022).

### DNA extraction

Specimens in ethanol were washed and rehydrated twice in 1x PBS for 20 min. To remove all the ethanol residues, they were dried for an additional 20 min. DNA was extracted from all ticks using DNeasy Blood & Tissue Kit (Qiagen, Hilden, Germany). Minor changes to the protocol were done according to the manufacturer instructions: proteinase K incubation was carried on overnight at 56 °C and DNA was eluted in two steps with 25 µL each of sterile water pre-heated to 72 °C. DNA was quantified and stored at −80 °C until use. The tick samples were pooled prior to sequencing to study the diversity of the microbial communities.

### Nested PCR

A total of 390 ticks were screened for SFGR and *Anaplasma* infections. The presence of SFGR was determined using *ompA* gene-specific primers (Mukherjee et al., 2014). For primary PCR, RR190-70 (50-ATGGCGAATATTTCTCCAAAA-30) and RR190-701 (50-GTTCCGTTAATGGCAGCATCT-30) primers were used; whereas for nested PCR, 190-FN1 (50-AAGCAATACAACAAGGTC-30) and 190-RN1 (50-TGACAGTTATTATACCTC-30) primers were used. 2.5 μL of DNA template was added to 8 μL of ultra-pure water, 12.5 μL of 2 × PCR Master Mix and 1 μL of each primer in the primary reaction. 2.5 μL of the primary PCR, 12.5 μL of 2 × PCR Master Mix, 1 μL of each nested primer and 8 μL of ultra-pure water were used in the nested reaction. PCRs were performed using the following sequence: 1 cycle at 95 °C for 3 min, 35 cycles at 95 °C for 20 sec, 46 °C for 30 sec and 63 °C for 60 s and 1 cycle at 72 °C for 7 min. DNA from *Rickettsia parkeri* was used as a positive control, and PCR mix with no DNA template was used as a negative control.

For detection of *Anaplasma*, we used EBR2, EHR 16SD and EBR3 primers. The EHR 16SD (5 – GGTACCYACAGAAGAAGTCC-3) primer amplified *16S* rDNA from all members of the Anaplasmataceae as described by Hornok et al. (2008). EBR2 (5 -TGCTGACTTGACATCATCCC-3) and EBR3 (5 – TTGTAGTCGCCATTGTAGCAC-3) primers were described by Teshale et al. (2015). We used EHR 16SD primer as a forward primer in the first round of amplification as it is already proved to be suitable for *Anaplasma* species determination and again used it for the second cycle of amplification as a forward external primer.

Nested PCR used EHR 16SD and EBR3 primers for the first round of PCR amplification to amplify a fragment of about 925 bp (16S rDNA gene). EHR 16SD was used as forward external primer, and EBR2 was used as internal primer for the second round of PCR amplification. Master mix was the same as used for *ompA*. The conditions for the first round were 92 **°**C for 3 min, 40 cycles of denaturation at 92 **°**C for 30 s, annealing for 45 s at 62 **°**C, elongation for 1 min at 72 **°**C and final extension for 10 min at 72 **°**C . PCR product (0.5 μL) from the first reaction was used as a template for the second round of amplification, which lasted for 25 cycles. Throughout the PCR procedures, negative samples were retested for any possible inhibition effect. DNA from *A. phagocytophilum* was used as a positive control, and PCR mix with no DNA template was used as a negative control.

### DNA sequencing and phylogenetic analysis

All the PCR products were examined by gel electrophoresis on 2% agarose gel containing ethidium bromide. PCR products (540 bp in length for *Rickettsia* and 925 bp for *Anaplasma*) were excised from the agarose gel, and then DNA extraction was done using a Monarch DNA gel extraction kit (New England BioLabs, Ipswich, MA, USA). The purified DNA samples were sent to Genomic Centre of University of Minnesota for Sanger sequencing. Gene amplicons were subjected to BLAST analyses for verification of *Rickettsia* and *Anaplasma* identification.

For both *Rickettsia* and *Anaplasma* PCR amplicons, Sanger sequence data were generated using forward and reverse primer pairs. Sequences were aligned and manually inspected using Geneious Prime (v2021.2.2) (Kearse et al., 2012). Pair-wise alignments were trimmed following visualization of each corresponding sequence chromatogram, and any sequences with poor chromatogram quality were excluded from downstream analyses. The resulting trimmed consensus sequences were aligned using MAAFT (v7.450) (Katoh and Standley, 2013).

Maximum likelihood phylogenies were generated using RAxML (v8.2.11) (Stamatakis, 2014) with the following parameters: rapid bootstrap analysis mode; 1000 bootstrap iterations; general time-reversible substitution model; and gamma model of rate heterogeneity (i.e. GTR + G). For the phylogenetic tree based on *Rickettsia ompA* gene, *Rickettsia tamurae* (AB114823) was used as an outgroup. Similarly, for the phylogeny based on *Anaplasma 16S* gene, *Ehrlichia ruminantium* (GenBank Accession no. DQ482915) was used as an outgroup. Phylogenetic trees were visualized and annotated using the Interactive Tree of Life online tool (v6.5.2, https://itol.embl.de/) (Letunic and Bork, 2024).

## Results

### Molecular detection of putative pathogenic spotted fever group of Rickettsia and Anaplasma

A total of 390 ticks collected from sheep, goats, cattle and buffaloes from different tick species, e.g. *Haemaphysalis punctata, H. sulcata, Hyalomma anatolicum, Hy. dromedarii, Hy. excavatum, Hy. rufipes, Hy. scupense, Rhipicephalus decoloratus, Rh. microplus* and *Rh. sanguineus*, were used in detection of SFGR and *Anaplasma*.

The RR190-70, RR190-701, 190-FN1 and 190-RN1 primers amplified parts of the rickettsial *ompA* gene. After amplification, bacterial DNA gave a clear single band at 540 bp. Positive samples comprised 7/390 (2.58%) belonging to 5 tick species. SFGR DNA was not amplified from *Hy. dromedarii, H. sulcata* and *Hy. rufipes*. According to nucleotide sequences, unknown *Rickettsia* sp. was detected in 14.28% (1/7) of *H. punctata* and uncultured *Rickettsia* sp. 2 was observed in 1.67% (1/60) of *Rh. sanguineus*, 1.09% (1/91) of *Hy. anatolicum* and 3.22% (1/31) of *Rh. microplus. Rickettsia* sp. 3 was detected in 1.56% (1/64) of *Hy. scupense.* Tick species positive SFGR and their prevalence are listed in Table 1.

**Table 1.** Putative pathogenic SFG Rickettsia (ompa) in tick species collected from variety of hosts

Tick species	Pathogen species	GenBank *ompA* Acc #	No. of ticks examined	No. of positive ticks (*ompA*)	Prevalence (%)
*Haemaphysalis punctata*	*Rickettsia* sp. 1	ON995346	7	1	14.28
*Rhipicephalus sanguineus*	*Rickettsia* sp. 2	ON995343	45	2	1.67
*Hyalomma anatolicum*	*Rickettsia* sp. 2	ON995348	125	2	1.09
*Rhipicephalus microplus*	*Rickettsia* sp. 2	ON995344	31	1	3.22
*Hyalomma scupense*	unknown *Rickettsia*	ON995347	63	1	1.56
Total number			271	7	2.58

The EBR2, EBR3 and 16SD primers amplified parts of the *Anaplasma* 16S rDNA gene. After amplification, bacterial DNA gave a clear single band at 925 bp. Among the tested ticks, 57/390 (14.62%) were positive for *Anaplasma* spp. DNA isolated from *Hy. dromedarii, H. punctata, H. sulcate and Hy. rufipes* did not amplify *Anaplasma. A. ovis* and *A. marginale* were detected in 32.25% of *Rh. microplus,* 20.63% of *Hy. scupense,* 20% of *Rh. sanguineus,* 6.25% of *Hy. excavatum* and 10.93% of *Hy. anatolicum* (Table 2).

**Table 2.** Putative pathogenic anaplasma (16S) in tick species collected from variety of hosts

Tick species	Pathogen species	GenBank 16S Acc #	No. of ticks examined	No. of positive ticks (16S)	Prevalence (%)
*Rhipicephalus sanguineus*	*Anaplasma ovis*	ON995350, ON995354, ON995356, ON995364, OP020282, OP020283, OP020300	45	7	20
	*Anaplasma marginale*	OP020270		1	
	*Anaplasma sp.*	OP020290		1	
*Hyalomma excavatum*	*Anaplasma ovis*	ON995351, OP020260, OP020264, OP020269, OP020297	80	5	6.25
*Hyalomma anatolicum*	*Anaplasma ovis*	ON995352, ON995362 OP020263, OP020267 OP020271, OP020274 OP020278, OP020279 OP020285, OP020293 OP020294, OP020299	128	12	10.93
	*Anaplasma marginale*	OP020280, OP020287		2	
*Rhipicephalus microplus*	*Anaplasma ovis*	ON995353, ON995355, ON995361, OP020265, OP020272, OP020276, OP020277, OP020288, OP020289	31	9	32.25
	*Anaplasma marginale*	OP020291		1	
*Hyalomma scupense*	*Anaplasma ovis*	ON995357, ON995360 ON995363, ON995365 OP020266, OP020268 OP020273, OP020275, OP020281, OP020284, OP020292, OP020295	63	12	20.63
	*Anaplasma marginale*	OP020298		1	
	*Anaplasma* sp. Pakistan	OP020286		1	
*Rhipicephalus decoloratus*	*Anaplasma ovis*	ON995358, ON995359	9	2	20
	*Anaplasma marginale*	OP020301		1	
*Hyalomma marginatum*	*Anaplasma ovis*	OP020261, OP020262, OP020296	20	3	15

### Phylogeny of bacteria

#### Phylogenetic tree based on *rickettsia ompa* gene

All tick sequences included in this study (GenBank accession nos ON995343–ON995349) formed three monophyletic clades. The first clade consists of two sequences (GenBank accession nos. ON995346 and ON995345) obtained from *H. punctata* and *Rh. sanguineus*, respectively, and are closely related to *Rickettsia japonica* and *Rickettsia heilongjiangensis*. A high bootstrap value of > 98 indicates that they belong to the same genotypes. Both of these sequences originated in China, and the hosts of both these samples were *Haemaphysalis* ticks.

The second cluster is formed by four sequences (GenBank accession nos. ON995343, ON995344, ON995348 and ON995349). Their strong relationship is supported by a high bootstrap value of 100. They are closely related to *Rickettsia rickettsii* (HM446486.1), *Rickettsia philipii* (EU109181) and *Rickettsia honei* (DQ309096). *Rickettsia rickettsii* (HM446486.1) and *R. philipii* (EU109181) were obtained from *Ambylomma americanum* and *Dermacentor occidentalis*, respectively, and the origin of both these sequences was the USA.

The sequence (GenBank accession no. ON995347) makes a distinct clade, supported by a high bootstrap value of 95. This sequence is genetically similar to the AB795207 sequence, originating from Uzbekistan. It also shares a close evolutionary relationship with another rickettsial sequence obtained from Madagascar from *Ambylomma variegatum* having GenBank accession no. KR492932.1. The tree is rooted by *R. tamurae*, which acts as an outgroup.

#### Phylogenetic tree based on *Anaplasma* 16S gene

The tree is well rooted by *Ehrlichia ruminatum,* which acts as an outgroup in this tree. The first ingroup, which is separated by a node having a bootstrap value of 100, is formed by *A. phagocytophilum* (GenBank accession nos. GU046565, AF481850 and AB196720). The ancestors for *A. platys* and *A. bovis* were recently separated from the cluster formed by the sequences included in this study. Tick sequences in this study formed two distinctive monophyletic clades with high bootstrap values. Both these sequences are grouped together, indicating they share similar genotypes. The first clade (GenBank accession nos. ON995350–ON995364) is genetically identical to *A. ovis* (EF587237) and *A. centrale* (AF309869) and share a common ancestor. *Anaplasma ovis* (EF587237) originated from China and is nestled between ON995350 and ON995364, while *A. centrale* (AF309869) is a vaccine strain obtained from Israel.

All the other 42 sequences from different tick species in this study (GenBank accession nos. OP020260–OP020301) were clustered together and phylogenetically related to *A. marginale* (GenBank accession nos. DQ341369 and DQ341370) from South China. Their close relation was supported statistically by a high bootstrap value of 99.

**Figure 1.**
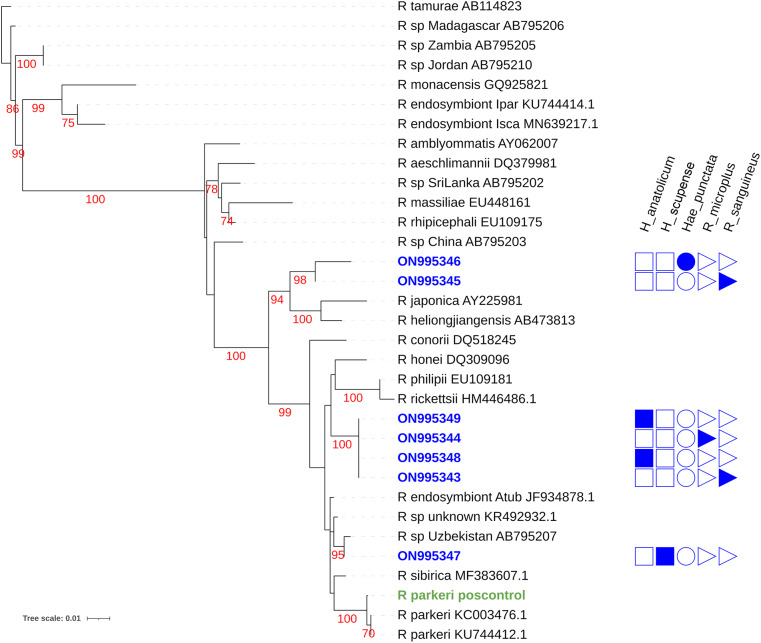
Maximum likelihood phylogenetic tree for the *Rickettsia ompa* gene for taxa detected in this study and others listed in NCBI genbank. Sequences obtained in this study are listed in blue as a genbank accession number with an indication of the tick species from which it was discovered. Red numbers indicate bootstrap values. Green text indicates the *R. parkeri* DNA positive control.

**Figure 2.**
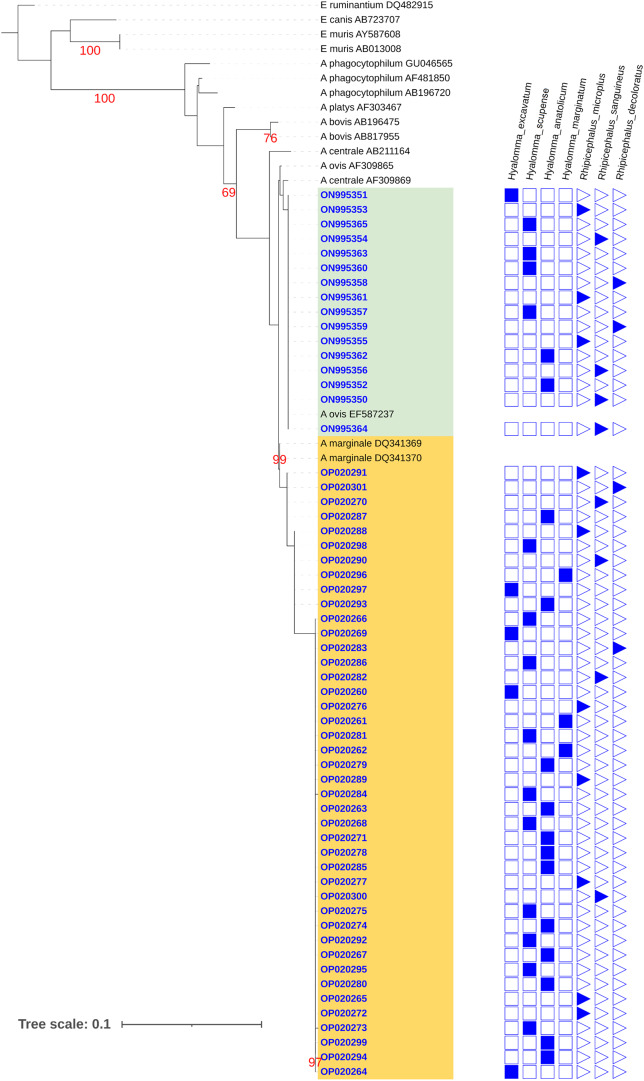
Maximum likelihood phylogenetic tree for the *Anaplasma 16S* rDNA gene for taxa detected in this study and others listed in NCBI genbank. Sequences obtained in this study are listed as a blue genbank accession number with an indication of the tick species from which it was discovered. Red numbers indicate bootstrap values. The green box indicates likely *A. ovis* sequences and the orange box indicates likely *A. marginale* sequences.

The phylogenetic trees of *Rickettsia* and *Anaplasma* are shown in Figure 1 and Figure 2.

## Discussion

SFGR are neglected diseases in developing countries across Asia and are emerging infections in all over the world (Chikeka and Dumler, 2015). The sub-tropical climate, humidity and high temperature result in the spread of ticks and tick-borne diseases in Pakistan, but the prevalence rates of these pathogens in ticks have not been much investigated (Ashraf et al., 2020). It is likely that a changing climate and associated changes in temperature and moisture patterns will affect tick distributions, phenology and disease transmission risks in unpredictable ways, further emphasizing the need for ongoing surveillance efforts (de Souza and Weaver, 2024).

SFGR and *Anaplasma* were detected by PCR amplification of *ompA* and *16S* gene. These genes have been used in previous studies to detect the presence of these pathogens in ticks (Baldridge et al., 2004; Teshale et al., 2015). Based on the single gene phylogeny (Figure 1), potentially novel SFGR were found in *Hy. scupense, H. punctata* and *R. sanguineus* ticks. Given the understudied nature of rickettsiae in Pakistan and global diversity of the clade, finding SFGR of uncertain identity is not surprising. Phylogenetic placement provides no evidence of whether these represent potential human or livestock pathogens. Further investigation of rickettsial distribution in ticks and associated livestock is warranted. Producing more complete genetic data to better define the phylogenic positions of potentially undescribed SFGR would also be of value in predicting risk of pathogenicity and better understanding SFGR biodiversity.

This study reported *Rickettsia* spp. in ticks collected from livestock. A study conducted in Khyber Pakhtunkhwa, Pakistan tested ticks collected from livestock and found 20.4% positivity for *Rickettsia* (Ali et al., 2022a). Another study conducted in the Federally Administered Tribal Areas of Khyber Pakhtunkhwa, Pakistan, reported prevalence of *Rickettsia massiliae* (42.6%)*, Rickettsia slovaca* (25.9%) and *Rickettsia conorii* (5.6%) in ticks collected from small ruminants (Ghafar et al., 2020), demonstrating that pathogenic SFGR are actively in circulation in Pakistan. In another study, SFGR-specific amplicons also were identified in 10% of ticks (514) collected from livestock, including from the potential pathogen *Rickettsia amblyommatis* (Karim et al., 2017).

Our study found *A. ovis* and *A. marginale* in tick samples collected from buffaloes, cattle, sheep and goats. Many previous studies have reported on bovine anaplasmosis-associated pathogens in large ruminants but *Anaplasma* prevalence in small ruminants needs more investigation (Zabel and Agusto, 2018). Abid *et al.* (2021) conducted a study in Layyah based on the detection of the *msp1b* gene in sheep and reported 6.9% of sheep as positive for *A. marginale.* Ghaffar et al. (2020) did a study in Mianwali, Pakistan, targeting *16S* rRNA gene and found many sheep (32%) positive for *A. marginale* and *A. ovis*. In other studies, 40% of sheep were positive for *A. marginale* on the basis of MSP5 indirect ELISA in Peshawar and 16.2% of sheep from Lakki Marwat and Peshawar were positive for *A. marginale* (Kashif and Ahmad, 2014; Turi, 2018). Hussain et al. (2017) reported that 42.7% of sheep in Karak, Khyber Pakhtunkhwa were positive for *A. marginale*. Many factors may affect the prevalence of *A. marginale* in sheep including habitat, tick control programmes, abiotic factors and the management methods of livestock farms (Belkahia et al., 2015).

The presence of SFGR and *Anaplasma* spp. in ticks collected from small ruminants represents a particular concern given the relative lack of research into this host/pathogen relationship. Sheep have been demonstrated to develop clinical illness when infected with *A. marginale* (Abdullah et al., 2020) and represent a likely reservoir for the infection of cattle. Further research is warranted to determine best practices in livestock husbandry to limit tick feeding on livestock, develop and distribute effective *A. marginale* vaccines to both cattle and small ruminants, and promote pasturage methods to limit contact between herds. More systematic surveillance of livestock infection, ticks and tick-borne pathogen diversity and biology will be required to accurately assess risk. Surveillance studies are particularly lacking in southern Pakistan where socioeconomic conditions are poorer and livestock rearing is more focused on smaller ruminants than on cattle. Improved surveillance, public education and research-informed policy development and enforcement will be needed to curb the spread of tick-borne livestock and human diseases in Pakistan.

## Data Availability

Sequencing data is available on National Center for Biotechnology Information GenBank (https://www.ncbi.nlm.nih.gov/genbank/).
